# Diffusion-based spatial priors for imaging

**DOI:** 10.1016/j.neuroimage.2007.07.032

**Published:** 2007-12

**Authors:** L.M. Harrison, W. Penny, J. Ashburner, N. Trujillo-Barreto, K.J. Friston

**Affiliations:** a The Wellcome Trust Centre for Neuroimaging, Institute of Neurology, University College London, 12 Queen Square, London, WC1N 3BG, UK; b Cuban Neuroscience Centre, Havana, Cuba

**Keywords:** Diffusion kernel, Weighted graph Laplacian, Spatial priors, Gaussian process model, fMRI, General linear model, Random effects analysis

## Abstract

We describe a Bayesian scheme to analyze images, which uses spatial priors encoded by a diffusion kernel, based on a weighted graph Laplacian. This provides a general framework to formulate a spatial model, whose parameters can be optimized. The application we have in mind is a spatiotemporal model for imaging data. We illustrate the method on a random effects analysis of fMRI contrast images from multiple subjects; this simplifies exposition of the model and enables a clear description of its salient features. Typically, imaging data are smoothed using a fixed Gaussian kernel as a pre-processing step before applying a mass-univariate statistical model (e.g., a general linear model) to provide images of parameter estimates. An alternative is to include smoothness in a multivariate statistical model (Penny, W.D., Trujillo-Barreto, N.J., Friston, K.J., 2005. Bayesian fMRI time series analysis with spatial priors. *Neuroimage* 24, 350–362). The advantage of the latter is that each parameter field is smoothed automatically, according to a measure of uncertainty, given the data. In this work, we investigate the use of diffusion kernels to encode spatial correlations among parameter estimates. Nonlinear diffusion has a long history in image processing; in particular, flows that depend on local image geometry (Romeny, B.M.T., 1994. Geometry-driven Diffusion in Computer Vision. Kluwer Academic Publishers) can be used as adaptive filters. This can furnish a non-stationary smoothing process that preserves features, which would otherwise be lost with a fixed Gaussian kernel. We describe a Bayesian framework that incorporates non-stationary, adaptive smoothing into a generative model to extract spatial features in parameter estimates. Critically, this means adaptive smoothing becomes an integral part of estimation and inference. We illustrate the method using synthetic and real fMRI data.

## Introduction

Functional MRI data are typically transformed to a three-dimensional regular grid of voxels in anatomical space, each containing a univariate time series of responses to experimental perturbation. The data are then used to invert a statistical model, e.g., general linear model (GLM), after a number of pre-processing steps, which include spatial normalization and smoothing (i.e., convolving the data with a spatial kernel). In mass-univariate approaches (e.g., statistical parametric mapping), a statistical model is used to extract features from the smoothed data by treating each voxel as a separate observation. Model parameters, at each voxel, are estimated (Friston et al., 2002) and inference about these parameters proceeds using SPMs or posterior probability maps (Friston and Penny, 2003). Smoothing the data ensures the maps of parameter estimates are also smooth. This can be viewed as enforcing a smoothness prior on the parameters. The current paper focuses on incorporating smoothness into the statistical model by making smoothness a hyperparameter of the model and estimating it using empirical Bayes. This optimizes the spatial dependencies among parameter estimates and has the potential to greatly enhance spatial feature detection.

Recently Penny et al. (2005) extended the use of shrinkage priors on parameter estimates (Penny et al., 2003), which assume spatial independence, to spatial priors in a statistical model of fMRI time series. They developed an efficient algorithm using a mean-field approximation within a variational Bayes framework. The result is a smoothing process that is incorporated into a generative model of the data, where each parameter is smoothed according to a measure of uncertainty in that parameter. The advantage of a mean-field approximation is that inversion of a requisite spatial precision matrix is avoided. The advantage of a Bayesian framework is that the evidence for different spatial priors can be compared (MacKay, 2003). Other Bayesian approaches to spatial priors in fMRI include those of Gossl et al. (2001); Woolrich et al. (2004); and more recently Flandin and Penny (2007).

There are two main departures from this previous work on spatiotemporal models in the current method. The first is that we use a Gaussian process prior (GPP) over parameter estimates. Spatial correlations are then encoded using a covariance matrix instead of precisions (cf. Penny et al., 2005). The second is that the covariance matrix is the Green's function of a diffusive process, i.e., a diffusion kernel, which encodes the solution of a diffusion equation involving a weighted graph Laplacian. This has the advantage of providing a full spatial covariance matrix and enables inference with regards to the spatial extent of activations. This is not possible using a mean-field approximation that factorizes the posterior distribution over voxels. The result is an adaptive smoothing that can be spatially non-stationary, depending on the data. This is achieved by allowing the local geometry of the parameter field to influence the diffusion kernel (smoothing operator). This is important as stationary smoothing reveals underlying spatial signal at the expense of blurring spatial features. Given the convoluted spatial structure of the cortex and patchy functional segregation, it is reasonable to expect variability in the gradient structure of a parameter field. The implication is that the local geometry of activations should be preserved. This can be achieved with a nonlinear smoothing process that adapts to local geometric ‘features’. A disadvantage is the costly operation of evaluating matrix exponentials and inverting potentially large covariance matrices, which the mean-field approach avoids. However, many approximate methods exist (MacKay, 2003; Rasmussen and Williams, 2006) that can ameliorate this problem, e.g., sparse GPPs (see discussion and Quinonero-Candela and Rasmussen, 2005).

The paper is organized as follows. First, we discuss background and related approaches, before giving an outline of the theory of the method. We start with the model, which is a two-level general linear model (GLM) with matrix-variate density priors on GLM parameters. We focus on reducing the model to the specification of covariance components, in particular, the form of covariance and its hyperparameters. We then look at the form of the spatial priors using graph Laplacians and diffusion kernels. We then describe the **EM** algorithm that is used to update hyperparameters of covariance components, which embody empirical spatial priors. The edge preserving quality of diffusion over a weighted graph is demonstrated using synthetic data and then applied to real fMRI data. The illustrations in this paper use 2D spatial images, however, the method can be easily extended to 3D, subject to computational resources, which would be necessary to analyze a volume of brain data. We perform a random effects (between subjects) analysis (Penny and Holmes, 2003) on a sample of contrast images from twelve subjects. This means that we consider a scalar field of parameter estimates encoding the population response. However, the nonlinear diffusion kernels described here can be extended to fields of vectors and matrices (ChefD'Hotel et al., 2004; Zhang and Hancock, 2006b). This paper concludes with comments on outstanding issues and future work.

## Background

The current work draws on two main sources in the literature; diffusion-based methods in image processing and Gaussian process models (GPM). The image processing community has been using diffusion models for many years, e.g., for the restoration of noisy images (Knutsson et al., 1983). For overviews, from the perspective of scale-space theories, see Romeny (1994, 2003). These models rest on the diffusion equation, which is a nonlinear partial differential equation describing the density fluctuations in an ensemble undergoing diffusion; *μ˙* = ∇·*D*(*μ*)∇*μ*, where *μ* can be regarded as the density of the ensemble (e.g., image intensity) and *D* is the diffusion coefficient. Generally, the diffusion coefficient depends on the density, however, if *D* is a constant, the equation reduces to the ‘classical heat equation’; *μ˙* = *D*∇^2^ *μ*, where ∇^2^ ≡ Δ is the Laplacian operator (second-order spatial derivative). A typical use in image processing is to de-noise an image, where the noisy image is the initial condition, *μ*(*t* = 0) and a smoothed, de-noised, image is the result of integrating the heat equation to evaluate the diffused image at some time later; *μ*(*t*). In particular, Perona and Malik (1990) used nonlinear diffusion models to preserve the edges of images using an image dependent diffusion term, *D* = *D*(∇*μ*). The dependence on this spatial gradient has the effect of reduced diffusion over regions with high gradient, i.e., edges. Later formulations of nonlinear diffusion methods include those of Alvarez et al. (1992) and Weickert (1996). Of particular relevance to the method presented here are graph-theoretic methods, which use graph Laplacians (Chung, 1991). These have been used recently to adaptively smooth scalar, vector and matrix-valued images (Zhang and Hancock, 2005). Graphical methods provide a general formulation on arbitrary graphs, which is easy to implement. There are also many useful graph-based algorithms in the literature, e.g., image processing on arbitrary graphs (Grady and Schwartz, 2003) and, more generally, graph partitioning to sparsify and solve large linear systems (Spielman and Teng, 2004).

Gaussian process models also have a long history. A Gaussian process prior (GPP) is a collection of random variables, any finite number of which have a joint Gaussian distribution (MacKay, 2003; Rasmussen and Williams, 2006). As such it is completely specified by a mean and covariance function. This is a very flexible prior as it is a prior over a function, which can be used to model general data, not just images. Given a function over space, this function is assumed to be a sample from a Gaussian random field specified by a mean and covariance, which can take many forms, as long as it is positive semi-definite.

Diffusion methods in image processing and covariance functions in GPMs furnish the basis of a spatial smoothing operator; however, the emphasis of each approach is different. One main difference is that a GPM is a statistical model from which inferences and predictions can be made (MacKay, 1998). The objective is not solely to smooth data, but to estimate an optimal smoothing operator, which is embedded in a model of how the data were generated. Graphical models in machine learning (Jordan, 1999) provide a general and easy formulation of statistical models. The similar benefits of graph-based diffusion methods in image processing further motivates the use of graph-theoretic approaches to represent and estimate statistical images, given functional brain data.

The relation between models of diffusion and GPPs is seen when considering a random variable as a diffusive process, which locally is a Gaussian process. We can see this by comparing the Green's function of the classical heat equation, used in early diffusion methods in image processing (Romeny, 1994) and the squared exponential (SE) covariance function used in GPMs (Rasmussen and Williams, 2006). In two dimensions, (*u*_*k*_, *u*_*l*_), where subscripts indicate location in the domain and *D* is a scalar;

(1) $$\dot{\mu }=D\Delta \mu$$

μ(t+τ)=K(τ)μ(t)

$$K(u_{k}\text{,}u_{l}\text{;}\tau )=\frac{1}{4\pi D\tau }\exp\left(-\frac{{\left(u_{k}-u_{l}\right)}^{T}(u_{k}-u_{l})}{4D\tau }\right)$$

where *K*(*τ*) is the Green's function (solution) of the diffusion equation that represents the evolution of a solution over time. The first line is the special case of constant diffusion coefficient. The solution of this equation is given in the second and third line, where the image at time *t*, *μ*(*t*), is propagated to *t* + *τ* by convolution with the Green's function, or practically by the matrix–vector product using the matrix exponential of the scaled discrete Laplacian. This Green's function is Gaussian with variance 2*Dτ*, meaning that the image at *t* + *τ* is a smoothed version of *μ*(*t*). This is shown explicitly in the last line^1^ , which has the same form as the SE covariance function, given below, where the squared characteristic length scale is *σ* ^2^ = 2*Dτ*. Typically, a GPP has an additional scale hyperparameter to give

(2) $$K(u_{k}\text{,}u_{l}\text{;}\lambda )=\upsilon \exp\left(-\frac{{\left(u_{k}-u_{l}\right)}^{T}(u_{k}-u_{l})}{2\sigma^{2}}\right)$$

where *λ* = (*υ*, *σ*). A zero mean GPP is then specified, at a set of locations, by the multivariate density, *μ* ∼ *N*(0, *K*) (Rasmussen and Williams, 2006). In what follows, we use a diffusion kernel as the covariance of a GPP. This is a spatial prior on model parameter images.

There are a number of papers applying methods from image processing to anatomical and functional brain images. These include those of Gerig et al. (1992), who applied nonlinear diffusion methods to MRI data and Chung et al. (2003) who used the Laplace–Beltrami operator (a generalization of the heat equation to a Riemannian manifold) in a statistical approach to deformation-based morphometry. Nonlinear diffusion methods have been used to adaptively smooth functional images (Kim and Cho, 2002; Kim et al., 2005). Other approaches to adaptive analysis of fMRI include those of Cosman et al. (2004); Friman et al. (2003); and Teo et al. (1997). Graph-theoretic approaches to image processing have been used to regularize diffusion tensor images (DTI) (Zhang and Hancock, 2006a). These authors used a weighted graph Laplacian, which is a discrete analogue of the Laplace–Beltrami operator, to adaptively smooth over a field of diffusion tensors, thereby preserving boundaries between regions, e.g., white matter tracts and grey matter.

**Fig. 1:**
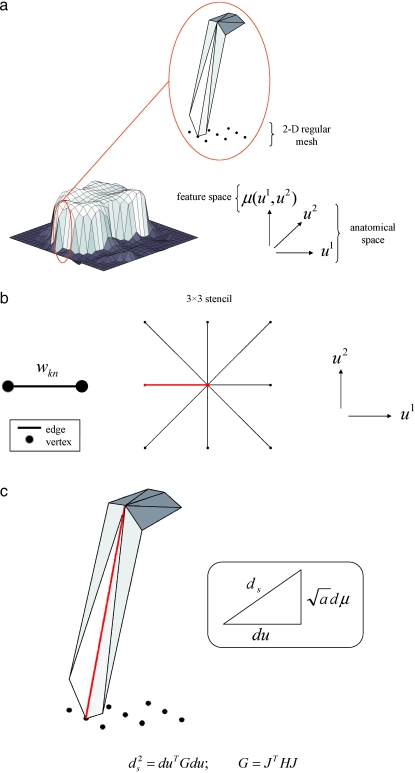
GLM parameters as a function over physical space: (a) parameter estimates, considered as a function, *μ*(*u*^1^, *u*^2^), over a 2D regular mesh, is thought of as a 2D sub-manifold embedded within a 3D space comprising ‘anatomical’ and ‘feature’ space coordinates. This is shown using parameter estimates from the synthetic data-set described in Fig. 4. The inset shows parameter estimates over a 3 × 3 stencil in physical space. Coordinates of the function in $R^{3}$ are (*u*^1^, *u*^2^, *μ*(*u*^1^, *u*^2^)). (b) A graph *Γ* is composed of vertices, edges and weights *Γ* = (*V*, *E*, *W*), shown for the 3 × 3 stencil in panel a, where the weight of an edge coupling the *k*^th^ and *n*^th^ vertices is *w*_*kn*_. (c) Graph weights are a function of distance, *d*_*s*_ (example shown in red and see Eq. (13)), on *μ*(*u*^1^, *u*^2^), such that *w*_*kn*_ ∈ [0, 1]. Note that the feature dimension is scaled by $\sqrt{a}$ (equivalent to ${\sqrt{a}}_{\mu }$ in main text), meaning that if *a* = 0 ⇒ *d*_*s*_ = d*u*, in which case the weights are independent of image intensity.

The contribution of our work is to combine graph-theoretic methods from image processing and Gaussian process models from machine learning to provide a spatial model of fMRI data. We are essentially constructing a manifold out of the parameter estimates of a linear model of fMRI data and performing isotropic diffusion on the induced manifold, which is anisotropic from the perspective of the domain. In other words, the diffusion is isotropic on the sub-manifold (that represents the surface of the image) embedded in anatomical–feature space (see Fig. 1 ), which is anisotropic in anatomical space. This is somewhat related to the random field approach (Worsley et al., 1999), where isotropic smoothing is attained by smoothing along an induced manifold. In our application we use anisotropic diffusion as an empirical spatial prior in a Bayesian setting.

## The model

In this section, we formulate a two-level GLM in terms of matrix-variate normal densities (Gupta and Nagar, 2000). Our focus is the formulation of this as a multivariate normal model, with emphasis on covariance components and their hyperparameters. We start with a linear model, under Gaussian assumptions, of the form

(3) $$\begin{array}{ccc} Y=X\theta +\varepsilon_{1} & & p(Y\text{,}\theta |X)=p(Y|X\text{,}\theta )p(\theta )\\ \theta =\varepsilon_{2} & \Rightarrow & p(Y|X\text{,}\theta )=\mathrm{MN}(X\theta \text{,}S_{1}⊗K_{1})\\ \varepsilon_{i}∼\mathrm{MN}(0\text{,}S_{i}⊗K_{i}) & & p(\theta )=\mathrm{MN}(0\text{,}S_{2}⊗K_{2}) \end{array}$$

where the left-hand expressions specify a hierarchical linear model and the right-hand expressions define the implicit generative density in terms of a likelihood, *p*(*Y*|*X*, *θ*) and prior, *p*(*θ*). MN stands for matrix-normal, where the density on matrix $A\in R^{r\times c}\text{,}A∼MN(M\text{,}S⊗K)$, has a mean, *M*, of size *r* × *c*, with covariances, *K* and *S*, of size *c* × *c* and *r* × *r*, that encode covariance between columns and rows respectively^2^ . Here, *Y* is a *T* × *N* data matrix and *X* is a *T* × *P* design matrix with an associated unknown *P* × *N* parameter matrix *θ*.

The errors at both levels have covariance *S*_*i*_ over rows (e.g., time, subjects or regressors) and *K*_*i*_ over columns (e.g., voxels). In this paper *S*_*i*_ are fixed. Eq. (3) is a typical model used in the analysis of fMRI data comprising *T* scans, *N* voxels and *P* parameters. The addition of the second level places empirical shrinkage priors on the parameters. This model can now be simplified by vectorizing each component using the identity vec(*ABC*) = (*C* ^*T*^ *⊗A*)vec(*B*) (see Appendix I and Harville, 1997)

(4) $$\begin{array}{ccc} y & = & Zw+e_{1}\\ w & = & e_{2}\\ e_{i} & ∼ & N(0\text{,}\Sigma_{i}) \end{array}$$

where *y* = vec(*Y*), *Z* = *I*_*N*_ *⊗X*, *w* = vec(*θ*), *e*_*i*_ = vec(*ε*_*i*_) and *Σ*_*i*_ = *K*_*i*_ *⊗S*_*i*_. *⊗* is the Kronecker product of two matrices and *I*_*N*_ is the identity matrix of size *N*. The unknown covariances *Σ*(*λ*)_1_ and *Σ*(*λ*)_2_ depend on hyperparameters, *λ*. The model parameters and hyperparameters are estimated using expectation maximization (**EM**) by maximizing the log-marginal likelihood

(5) $$\begin{array}{c} \ln p(y|\lambda )\geq F=-\frac{1}{2}(\ln|\Sigma |+y^{T}\Sigma^{-1}y+TN\ln2\pi )\\ \Sigma (\lambda )=\Sigma_{1}+Z\Sigma_{2}Z^{T} \end{array}$$

with respect to the parameters in the **E**-Step and the covariance hyperparameters in the **M**-Step. Here, *Σ*(*λ*) represents the covariance of the data induced by both levels of the model. The model inversion with **EM** will be described later (see also Appendices I and II). First, we look at the hyperparameterization of the spatial covariances and the specific forms of *K*(*λ*) entailed by *Σ*_*i*_ = *K*_*i*_ *⊗S*_*i*_.

## The priors

In the previous section, we reduced the problem of specifying a linear empirical Bayesian model to the specification of prior covariance components for noise and signal. In this section, we introduce diffusion-based spatial priors and consider adaptive priors that are functions of the parameters. In brief, we will assume the error or noise covariance is spatially unstructured; i.e., *Σ*_1_ = *K*_1_ *⊗S*_1_, where *K*(*λ*)_1_ = *υ*_1_ *I*_*N*_ and *S*_1_ = *I*_*T*_. This means that *υ*_1_ is the error variance. For simplicity, we will assume that this is fixed over the same for all voxels; however, it is easy to specify a component for each voxel, as in conventional mass-univariate analyses.

For the signal, we adopt an adaptive prior using a non-stationary diffusion kernel, which is based on a weighted graph Laplacian (see Chung, 1991 and next section), *L*(*μ*), which is a function of the conditional expectation of the parameters, *μ* = 〈*w*〉

(6) $$\begin{array}{c} K{\left(\lambda \right)}_{2}=\upsilon_{2}K_{\text{D}}\\ K_{\text{D}}=\exp(-L(\mu )\tau ) \end{array}$$

This means the hyperparameters comprise, *λ* = {*υ*_1_,*υ*_2_,*τ*}, where the first hyperparameter controls a stationary independent and identical (i.i.d.) noise component, the second the amplitude of the parameter image and third its dispersion. The matrix *L* in Eq. (6) is a weighted graph Laplacian, which is a discrete analogue of the Laplace–Beltrami operator used to model diffusion processes on a Riemannian manifold. The solution of the heat equation is^3^

(7) $$\dot{\mu }=-L(\mu )\mu \Rightarrow$$

μ(t+τ)=K(μ(t),τ)μ(t)

K(μ(t),τ)=exp(−L(μ)τ)

The diffusion kernel, *K* = exp(− *Lτ*), is the local solution to the heat equation on a graph and corresponds to a symmetric diffusion kernel that encodes the dispersion of *μ*, over a period *τ*. The diffusion kernel also evolves according to the heat equation

(8) $$\begin{array}{ccc} \dot{K} & = & -LK\\ K(t+\tau ) & =\exp(-L\tau )K(t) \end{array}$$

We use this diffusion kernel as the covariance matrix of a GPM. Generally, the Laplacian is a function, *L*(*μ* ^(*m*)^), of the current image (of parameter expectations), where the superscript indicates the *m* ^th^ iteration. In this situation, Green's function is a composition of local solutions.

(9) $$\begin{array}{ccc} K{\left(\lambda \right)}_{2} & = & \upsilon_{2}K_{\text{D}}\\ K_{\text{D}}^{\left(m\right)} & = & P^{\left(m\right)}P^{\left(m-1\right)}\ldots P^{\left(1\right)}=P^{\left(m\right)}K_{\text{D}}^{\left(m-1\right)}\\ P^{\left(m\right)} & = & \exp(-\tau^{\left(m\right)}L^{\left(m\right)}) \end{array}$$

Updating *K*_D_^(*m*)^ requires computation of the current Laplacian *L* ^(*m*)^ and a matrix multiplication, both of which are costly operations on large matrices. However, if the Laplacian is approximately constant then *K*_D_^(*m*)^ can be evaluated much more simply

(10) $$\begin{array}{ccc} K_{\text{D}}^{\left(m\right)} & = & P^{\left(m\right)}P^{\left(m-1\right)}\ldots P^{\left(1\right)}\\ & \approx & \exp(-(\tau^{\left(m\right)}+\tau^{\left(m-1\right)}+\ldots +\tau^{\left(1\right)})L)=\exp(-L\tau ) \end{array}$$

This approximation retains the edge preserving character of the diffusive flow, without incurring the computational cost of re-evaluating the Laplacian. Our experience is that weighted graph Laplacians based on the ordinary least squares (OLS) estimate, *μ*_ols_, gives very reasonable results. However, the need to update the Laplacian may arise when the OLS parameter estimate is very noisy. All anisotropic Laplacian priors in this paper are based on *μ*_ols_; however, we have included update equations based on the Baker–Campbell–Hausdoff formula in Appendix IV.

In summary, the covariance components and their derivatives are:

(11) $$K_{1}=\upsilon_{1}I_{N}$$

$$K_{2}=\upsilon_{2}\exp(-L(\mu_{\text{ols}})\tau )$$

$$\partial K_{1}/\partial \upsilon_{1}=I_{N}$$

$$\partial K_{2}/\partial \upsilon_{2}=K_{\text{D}}$$

$$\partial K_{2}/\partial \tau =-\upsilon_{2}LK_{\text{D}}$$

where their hyperparameters *λ* = {*υ*_1_, *υ*_2_, *τ*} are optimized to ensure an optimal balance between signal and noise and that the parameter estimates have an optimal, non-stationary and non-isotropic smoothness encoded by the spatial covariance, *K*_2_. In the next section, we review graph Laplacians and the diffusion model in more detail and then conclude with a summary of the **EM** scheme used for optimization.

## Diffusion on graphs

In this section, we describe diffusion on graphs and illustrate how this furnishes useful spatial priors for parameters at each voxel. This formulation is useful as it is easily extended to vector and matrix-valued images, which will be necessary when modeling a general vector-field of parameter estimates, e.g., when the number of columns of the design matrix is greater than one. We start with some basic graph theory and then discuss diffusion in terms of graph Laplacians. The end point of this treatment is the form of the diffusion kernel, *K*_D_, used in the spatial prior of the previous section. We will see that this is a function of the parameters that enables the prior smoothness to adapt locally to non-stationary features in the image of parameter estimates.

### GLM parameters as a function on a graph

We consider the parameter estimates as a function on a graph, *Γ*, with vertices, edges and weights, *Γ* = (*V*, *E*, *W*). The vertices are indexed 1 to *N* and pairs are connected by edges, *E*_*kn*_, where (*k*, *n*) ∈ *V*. If two vertices are connected, i.e., are neighbors, we write *k* ∼ *n*. Consider a regular 2D mesh with spatial coordinates *u* ^1^ and *u* ^2^. Fig. 1a shows a surface plot of OLS parameter estimates of synthetic data described later (see Fig. 4) to illustrate the construction of a weighted graph Laplacian. To simplify the discussion we concentrate on a small region over a 3 × 3 grid, or stencil (see inset). Pairs of numbers *u* ^1^, *u* ^2^ indicate a vertex or pixel location, where each number corresponds to a spatial dimension. The function has a value at each pixel (voxel if in 3D anatomical space) given by its parameter estimate *μ*(*u*), so that three numbers locate a pixel at which a parameter has a specific value, *u* ^1^, *u* ^2^, *μ*(*u* ^1^, *u* ^2^). These are coordinates of the parameter estimate at a pixel in Euclidean space, $R^{3}$, which decomposes into ‘anatomical’ and ‘feature’ space coordinates (lower right of Fig. 1a). In this case these are 2 and 1 respectively. The 2D image is considered as a 2D sub-manifold of this 3D embedding space (Sochen et al., 1998), which provides a general framework that is easily extended to 3D anatomical space and feature dimensions greater than one. We represent the *k* ^th^ pixel by *v*_*k*_. Distance between two pixels is taken as the shortest distance along the 2D sub-manifold of parameter estimates embedded in $R^{3}$. This is a geodesic distance between points on the sub-manifold, *d*_*s*_(*v*_*k*_, *v*_*n*_). This is shown schematically in Fig. 1c between neighboring pixels. The shortest distance is easy to compute for direct neighbors (example shown in red), however, if the stencil were larger then fast marching algorithms (Sethian, 1999) may be used to compute the shortest path between two points on the sub-manifold. Note that the displacement along the feature coordinates is scaled by $\sqrt{a}$, such that if *a* = 0, then *d*_*s*_ is reduced to distance on the 2D domain and is no longer a function of image intensity (see subsection on special cases). The construction of a weighted graph Laplacian starts by specifying weights of edges between vertices, *w*_*kn*_. These are a function of the geodesic distance, *d*_*s*_(*v*_*k*_, *v*_*n*_), and are important for specifying non-stationary diffusion. This is shown in Fig. 1b for the 3 × 3 stencil in Fig. 1a.

### Graph Laplacian

As mentioned above, a graph is composed of vertices, edges and weights. Neighboring vertices are encoded by the adjacency matrix, *A*, with elements

(12) $$a_{kn}=\left\{ \begin{array}{c} 1\;\text{for}\;k∼n\\ 0\;\text{otherwise} \end{array}\right.$$

Weights make up a weight matrix, *W*, with elements

(13) $$w_{kn}=\left\{ \begin{array}{c} \exp(-d_{s}{\left(v_{k}\text{,}v_{n}\right)}^{2}/\kappa )\;\text{for}\;k∼n\\ 0\;\text{otherwise} \end{array}\right.$$

The un-normalized Laplacian of *Γ* is *L* = *D* − *W*, where *D* is a diagonal matrix with elements *D*_*kk*_ = *Σ*_*n*_ *w*_*kn*_, which is the degree of the *k* ^th^ vertex. The graph Laplacian is sometimes called the admittance or Kirchhoff matrix. The weights *w*_*kn*_ ∈ [0, 1] encode the relationship between neighboring pixels and are symmetric; i.e., *w*_*kn*_ = *w*_*nk*_. They play the role of conductivities, where a large value enables flow between pixels. *κ* is a constant that controls velocity of diffusion, which we set to one.

The weights are a function of the distance, *d*_*s*_(*v*_*k*_, *v*_*n*_), on the surface of the function, *μ*(*u*), between vertices *v*_*k*_ and *v*_*n*_. It is this distance that defines the nature of diffusion generated by the weighted graph Laplacian. In brief, we will define this distance to make the diffusion isotropic on the surface of the parameter image. This means, when looked at from above, that the diffusion will appear less marked when the spatial gradients of parameters are high. In other words, diffusion will be attenuated at the edges of regions with high parameter values. More formally, we specify the distance by choosing a map, *χ*, from the surface of the function *μ*(*u*) to an embedding space, the Euclidean space of $R^{3}$. Each space has a manifold and metric, (*M*, *g*) and (*N*, *h*), respectively (see Appendix III for more details and a heuristic explanation).

(14) $$\begin{array}{l} \chi :M\to N\\ \chi :u\to (\chi^{1}(u)\text{,}\chi^{2}(u)\text{,}\chi^{3}(u))=(u^{1}\text{,}u^{2}\text{,}\mu (u^{1}\text{,}u^{2})) \end{array}$$

Choosing a metric, *H*, of the embedding space (see below) and computing the Jacobian, *J*, we can calculate the induced metric, *G*, on *μ*(*u*) (Sochen et al., 1998). In matrix form, these are

(15) $$H=\left(\begin{array}{ccc} a_{1} & 0 & 0\\ 0 & a_{2} & 0\\ 0 & 0 & a_{\mu } \end{array}\right)J=\frac{\partial \chi }{\partial u}=\left(\begin{array}{cc} 1 & 0\\ 0 & 1\\ \mu_{\mu^{1}} & \mu_{\mu^{2}} \end{array}\right)$$

where *a*_*i*_ are the relative scales among dimensions and derivatives are with respect to physical space; i.e., *μ*_*x*_ = ∂*μ*/∂*x*, which are computed using central differences. The induced metric is then

(16) $$G=J^{T}HJ=\left(\begin{array}{cc} a_{1}+a_{\mu }\mu_{u^{1}}^{2} & a_{\mu }\mu_{u^{1}}\mu_{u^{2}}\\ a_{\mu }\mu_{u^{1}}\mu_{u^{2}} & a_{2}+a_{\mu }\mu_{u^{2}}^{2} \end{array}\right)$$

which is used to calculate distance

(17) $$d_{s}^{2}=du^{T}Gdu$$

where d*u* = (d*u* ^1^, d*u* ^2^)^*T*^. Due to the dependence of the graph Laplacian on the parameters we write the Laplacian as *L* = *L*(∇*μ*_ols_), where the Laplacian is computed using the OLS estimate, *μ*_ols_. As this depends on geodesic distances on the embedded sub-manifold of an image we call it a geodesic graph Laplacian (GGL). If *a*_*μ*_ = 0 then the Laplacian is based on Euclidean distance in anatomical space. We refer to this as a Euclidean graph Laplacian (EGL) (see also subsection below on special cases). Note that we have chosen the embedding coordinates and embedding space metric. This is one of the advantages of a geometric formulation as we could have chosen a non-Euclidean anatomical space, e.g., spherical coordinates to model diffusion on the surface of a sphere (see Sochen et al., 2003; Sochen and Zeevi, 1998). The diffusion kernel can be computed efficiently using an eigenvalue decomposition of the Laplacian.

(18) $$\begin{array}{ccc} L & = & V\Lambda V^{T}\\ \text{diag}(\Lambda ) & = & (\lambda_{0}\text{,}\lambda_{1}\text{,}\ldots \text{,}\lambda_{N-1})\text{,}\;\lambda_{i}\geq 0\\ K & = & V\exp(-\Lambda )V^{T} \end{array}$$

This is a standard method for computing the matrix exponential (Moler and Van Loan, 2003) with the added benefit that knowing the eigensystem simplifies many computations in the algorithm. See Appendix IV for more details.

## Expectation maximization

Inversion of the multivariate normal model in Eq. (4) is straightforward and can be formulated in terms of expectation maximization (**EM**). **EM** entails the iterative application of an **E**-Step and **M**-Step (see Appendix 1 and Friston et al., 2002, 2007 for details). The **E**-Step evaluates the conditional density of the parameters in terms of their expectation and precision (i.e., inverse variance); *p*(*w*|*y*,*λ*) = *N*(*μ*, *Π* ^− 1 ^), where

(19) $$E\text{−Step}\;\begin{array}{ccc} \mu & = & \prod^{-1}Z^{T}\Sigma_{1}^{-1}y\\ \prod & = & \Sigma_{2}^{-1}+Z^{T}\Sigma_{1}^{-1}Z \end{array}$$

The unknown covariances *Σ*(*λ*)_*i*_ = *K*_*i*_ *⊗S*_*i*_ are functions of covariance hyperparameters, *λ*. These are estimated by maximizing the log-marginal likelihood, ln *p*(*y*|*λ*), in an **M**-Step. This involves updating the hyperparameters by maximizing a bound on the log-marginal likelihood or log-evidence

(20) $$\ln p(y|\lambda )\geq F=-\frac{1}{2}(\ln|\Sigma |+y^{T}\Sigma^{-1}y+TN\ln2\pi )$$

We update hyperparameters (indexed by subscripts) using a Fisher-scoring scheme^4^ , where Δ*λ* represents incremental change of *λ*

(21) $$M\text{−Step}\;\begin{array}{ccc} \Delta \lambda & = & I{\left(\lambda \right)}^{-1}\nabla_{\lambda }F\\ \frac{\partial F}{\partial \lambda_{k}} & = & -\frac{1}{2}tr\left(\Sigma^{-1}\frac{\partial \Sigma }{\partial \lambda_{k}}\right)+\frac{1}{2}y^{T}\Sigma^{-1}\frac{\partial \Sigma }{\partial \lambda_{k}}\Sigma^{-1}y\\ I_{kl} & = & -\langle \frac{\partial^{2}F}{\partial \lambda_{k}\partial \lambda_{l}}\rangle =\frac{1}{2}tr\left(\Sigma^{-1}\frac{\partial \Sigma }{\partial \lambda_{k}}\Sigma^{-1}\frac{\partial \Sigma }{\partial \lambda_{l}}\right) \end{array}$$

*I*(*λ*) is the expected information matrix, see Wand (2002), with element *I*_*kl*_, where the expectation, 〈〉, is over the marginal likelihood of the data, ∇*_λ_F* is the score, i.e., a vector of gradients (*k* ^th^ element given by ∂*F*/∂*λ*_*k*_) with respect to the hyperparameters and *Σ* is the current maximum likelihood estimate of the data covariance (see Appendix I). In the examples below, we fix *S*_1_ = *I*_*T*_ and *S*_2_ = 1; this means the only unknown covariances are *K*(*λ*)_*i*_. This scheme is formally identical to classical restricted maximum likelihood (ReML) (see Friston et al., 2007).

In summary, to invert our model we simply specify the covariances *K*(*λ*)_*i*_ and their derivatives, ∂*K*/∂*λ*_*i*_. These enter an **M**-Step to provide ReML or ML-II estimates of covariance hyperparameters. *K*(*λ*)_*i*_ are then used in the **E**-Step to provide the conditional density of the parameters. **E**- and **M**-Steps are iterated until convergence, after which, the objective function for the **M**-Step can be used as an approximation to the models log-evidence. This quantity is useful in model comparison and selection, as we will see later when comparing models based on different spatial priors.

**Fig. 2:**
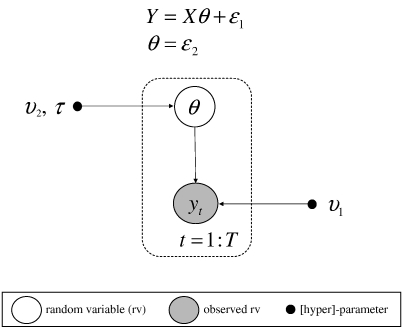
Generative model: single observations of an image are generated from the GPP, *p*(vec(*θ*)|*λ*) = *N*(0, *Σ*_2_), parameterized by *υ*_2_,*τ*. Multiple observations are collected in the matrix *Y*. *X* is the design matrix and the noise model is an i.i.d. Gaussian distribution, *p*(vec(*ε*_1_)) = *N*(0,*υ*_1_*I*_*TN*_).

We now have all the components of a generative model (shown schematically in Fig. 2 ) that, when inverted, furnishes parameter estimates that are adaptively smooth, with edge preserving characteristics. Furthermore, this smoothing is chosen automatically and optimizes the evidence of the model. Before applying this scheme to synthetic and real data we will consider some special cases that will be compared in the final section.

## Special cases

### Linear diffusion: *a*_*μ*_ = 0

If we set the scale of the parameter dimension *a*_*μ*_ = 0 (see Eq. (16) and Fig. 1c) we recover linear diffusion. The Laplacian (EGL) is now independent of *μ*. In this case edges are not preserved by the smoothness prior. Although these kernels are not the focus of this paper they are still useful and, as we demonstrate later, produce compelling results compared to non-spatial priors.

### Global shrinkage priors: *K*_D_ = *I*

If we removed diffusion by setting the Laplacian to zero then *K*_2_ = *υ*_2_ *I*_*N*_. This corresponds to global or spatially independent (shrinkage) priors (GSP) of the sort used in early posterior probability maps using empirical Bayes (Friston and Penny, 2003). Here, we use the variability of parameter estimates over pixels to shrink their estimates appropriately and provide a posterior or conditional density.

### Ordinary least squares estimate: *K*_2_ = 0

The OLS estimate obtains when we remove the empirical priors completely by setting *K*_2_ = 0.

## Synthetic data: de-noising an image

**Fig. 3:**
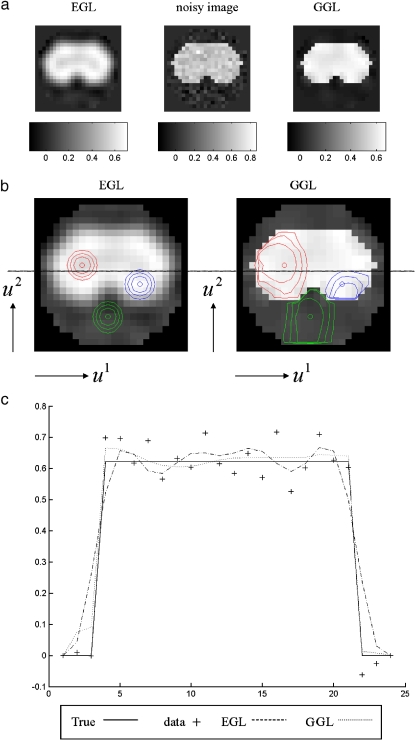

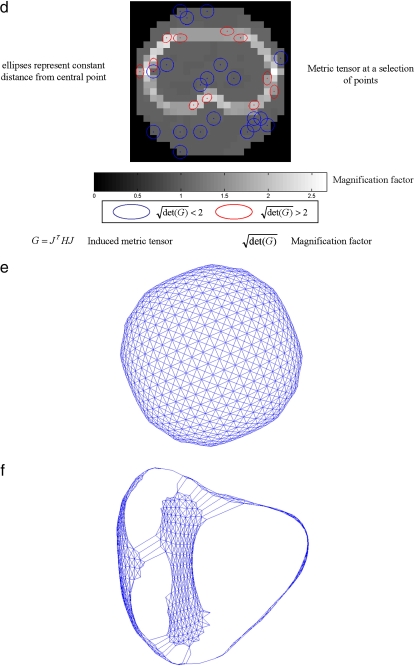
Synthetic data — de-noising an image: (a) a binary image with added noise is shown in the central panel (see grey-scale for pixel values). GPMs using diffusion kernels from EGL and GGL are shown on left and right respectively. On the left, noise is removed along with the edge of the original image. On the right, noise has been removed, while preserving the edge of the original object. (b) The left and right images of (a) with contour plots of local diffusion kernels from three locations overlaid. These are the same for kernels from EGL; however, is not the case for GGL. Local kernels adapt to the edge of the central image. (c) Cross-section at the level indicated in panel b. This shows the original binary image, noisy image, smoothing with EGL and GGL. For GGL, the noisy image is preferentially smoothed on flat regions. (d) Two representations of the induced metric tensor, *G*; square-root of determinant (area ratio of surface:domain) and orientation/magnitude (eigenvectors/values). Large ratio and aligned, smaller ellipses at the image edge is associated with reduced flow across the edge. (e and f) Global property of EGL and GGL shown using gplot.m (Matlab routine) of second and third eigenvectors of graph Laplacians. Symmetry is due to circular domain of image.

In this section, we apply the algorithm to one image, i.e. *T* = 1, to demonstrate edge preservation and provide some intuition as to how this is achieved using a diffusion kernel based on a GGL and compare it to EGL. We use synthetic data shown in Fig. 3 . The model is

(22) $$\begin{array}{ccc} y & = & w+e_{1}\\ w & = & e_{2} \end{array}$$

The central panel of Fig. 3a contains a binary image of a 2D closed curve (with values equal to one within and on the curve) on a circular background of zeros. Gaussian noise has been added, which we will try to remove using a GPM based on a diffusion kernel. The left panel shows the conditional expectation or mean using a diffusion kernel from a EGL, while GGL is shown on the right. Below each image is a color bar, which encodes the grey-scale of each pixel in the image. It is immediately obvious that smoothing with a EGL removes noise at the expense of blurring the image, while the GGL preserves edges. This is reflected in the values of log-marginal likelihood achieved for each prior (see Table 1 ). We will discuss the ratio of these values in the next section when we consider model comparison.

Fig. 3b shows contours of local diffusion kernels at three locations in the image, where the local diffusion kernel at the *k* ^th^ pixel is a 2D image reconstructed from the appropriate row of *K*_2_. These are superimposed on the smoothed image. Locations include (i) within the perimeter of the object, (ii) near the edge inside the perimeter of the object and (iii) outside the perimeter of the object. The EGL is on the left, where local kernels are the same through-out the image. This is not the case for the GGL on the right. The EGL smoothes the image isotropically, i.e., without a preferred direction. On the right, local kernels within and outside the perimeter of the object are different, depending on their relation to the edge of the central object. The contours of kernels within the perimeter spread into the interior of the object, but stop abruptly at the edge, encoding the topology of the surface, much like the contours on a geographic map. As a result the image is smoothed such that noise is removed, but not at the expense of over smoothing the edges of signal. This is shown in Fig. 3c for a cross-section at the level indicated by a dotted line in Fig. 3b. This shows the original binary image, noisy image, smoothing with EGL and GGL.

Further intuition comes from considering the induced metric tensor, *G*; consider the square-root of the determinant, $\sqrt{\det(G)}$, which is the ratio of surface and domain areas. An area element on the surface of *μ*(*u*) is $dA_{s}=\sqrt{\text{det}(G)}du^{1}du^{2}$, while one on the domain is d*A*_*D*_ = d*u* ^1^d*u* ^2^. This gives a ratio $dA_{S}/dA_{\text{D}}=\sqrt{\text{det}(G)}$, which can be calculated at each location of the image. This is referred to as the magnification factor (Bishop, 1999). This provides a scalar value at each pixel that represents a salient difference between the sub-manifold of the function, compared to the flat surface of the domain. This is shown in Fig. 3d. Flat regions have ratio of about one, while edges are greater than unity. High values correspond to locations where the distance on *μ*(*u*) between adjacent pixels (see Fig. 1) is large; i.e., at an edge, where gradients are large. This results in a small weight across the edge connecting these pixels and reduced flow. The effect is that regions with large gradients have less smoothing. As large gradients are a feature of edges, this means that they are preserved. To highlight the anisotropic nature of the ensuing diffusion, we have super-imposed ellipses representing the orientation and magnitude (eigenvectors and eigenvalues respectively) of *G* at a selection of different locations. Red and blue ellipses represent $\sqrt{\text{det}(G)}$ greater and lower than 2, respectively. It can be seen that the metric tensor is aligned with the edge of the central figure and isotropic elsewhere. This leads to preferential diffusion within the image and edge preservation.

Lastly, we have included a representation of a global property of the graph Laplacian using the graph plot routine in Matlab (gplot.m) of the second and third eigenvectors (of the Laplacian) in Figs. 3e and f. This is a standard representation of similarity between vertices on a graph. The second eigenvector is known as the Fiedler vector (Chung, 1991) and is used in graph-partitioning algorithms. The EGL is regular, whereas the GGL illustrates partitioning of the image into two distinct parts; the central region, which represents the central object in the image of Fig. 3a, while the background is represented by the periphery of the plot.

## Evaluations

In this section, we compare the performance of three different Gaussian process priors used to model the same data. These were global shrinkage priors (GSP) and diffusion kernels from Euclidean (EGL) and geodesic graph Laplacians (GGL).

### Synthetic data: random effects analysis

**Fig. 4:**
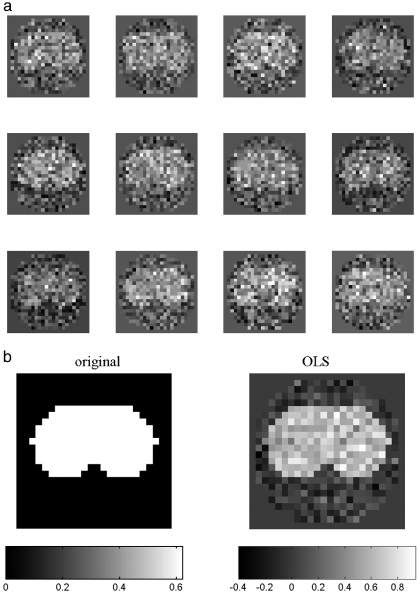

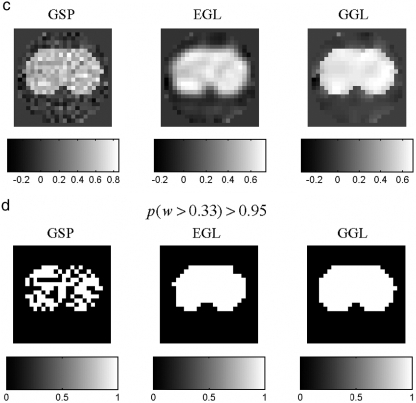
Synthetic data — random effects analysis: (a) twelve samples of the binary image shown in Fig. 3, (b) original image and OLS estimate, (c) estimated conditional means using GSP, EGL and GGL, (d) posterior probability maps at thresholds, *p*(*w* > 0.33) > 0.95.

The next simulation is similar to the above except now we have twelve samples of the image. Their purpose is to demonstrate posterior probability maps (PPMs) and model selection. The samples, original image, OLS estimate and estimated posterior means are shown in Figs. 4a–c. Fig. 4d compares PPMs using the three different priors. The first observation is enhanced detection of the signal with EGL and GGL compared to GSP. The second is the edge preserving nature of GGL. The evidence for each model is shown in Table 2 . As expected, given data with edges, the GGL attains the highest evidence. The log-marginal likelihood ratio (natural logarithm) for EGL and GGL was 146, which is very strong evidence in favor of the GGL. A direct comparison of the log-marginal likelihood is possible as the number of hyperparameters is equal for EGL and GGL. Additional penalty terms can be included for model comparison based on the number of hyperparameters used in a model and their uncertainty (Bishop, 1995). Details of these additional terms are included in Appendix II.

### Real data: random effects analysis

**Fig. 5:**
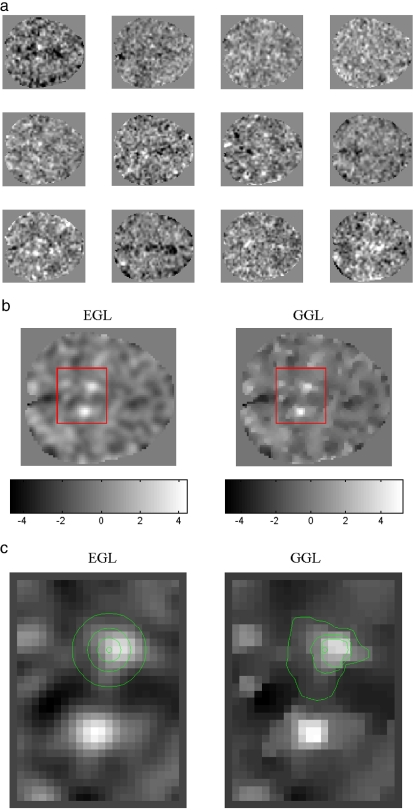

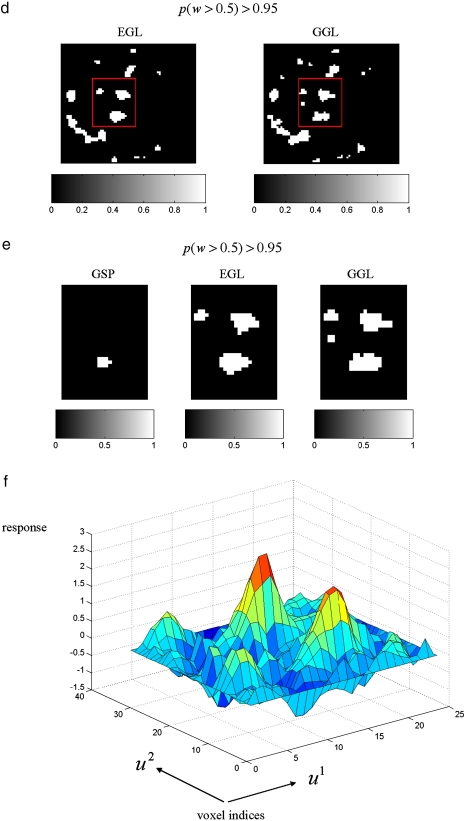

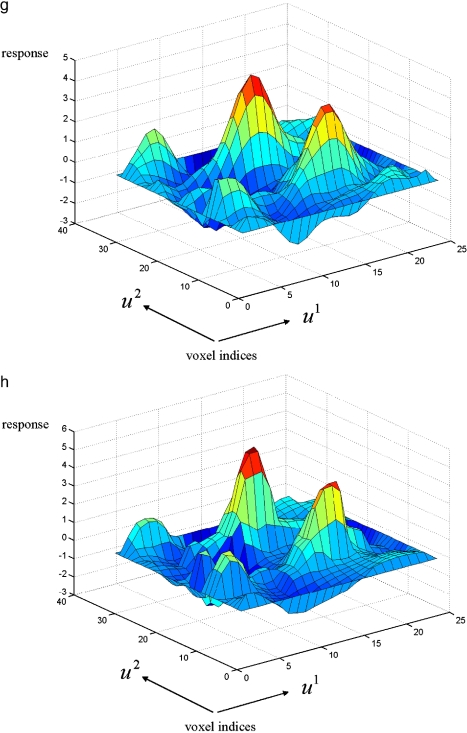

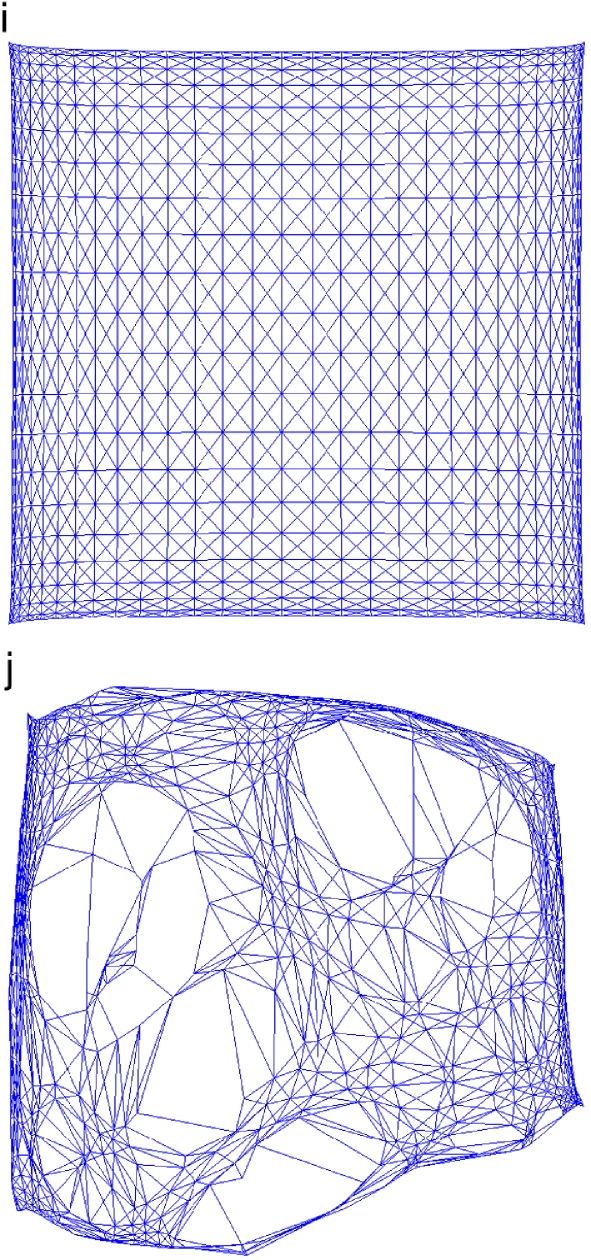
Real data: twelve contrast images of a slice showing bilateral response in posterior cingulated gryi (pCG) during a study of coherent motion (Harrison et al., 2007). (a) Twelve samples (b) estimated conditional means using EGL and GGL (left and right). (c) Inset of panel b with contour plot of a local diffusion kernel overlaid. Distinguishing borders between regions of high/low parameter estimates is difficult due to smoothing by the EGL. However, borders are easily seen on the right. (d) Posterior probability maps; where white regions indicate *p*(*w* > 0.5) > 0.95. (e) Inset of panel d for GSP, EGL and GGL. Active regions using EGL are characterized by rounded edges, i.e., blobs, while for GGL the shape of bilateral response are elongated in fitting with the anatomy of pCG, (f–h) surface plots of conditional means from inset. Note vertical scale, especially for GSP, which shows large shrinkage compared to EGL and GGL, (i and h) graph plot (gplot.m) of second and third eigenvectors of EGL and GGL. Heterogeneous graph weights of GGL are easily observed compared to EGL.

fMRI data collected from twelve subjects during a study of the visual motion system (Harrison et al., 2007) were used for our comparative analyses. The study had a 2 × 2 factorial design with motion type (coherent or incoherent) and motion speed as the two factors. Single subject analyses were performed, with no smoothing, using SPM2 (http://www.fil.ion.ucl.ac.uk/spm) to generate contrast images of the main effect of coherence. Images (one slice) of the twelve contrast images are shown in Fig. 5a. These constitute the data, *Y,* and the design matrix, *X* = 1, was a column of ones, implementing a single-sample *t*-test. The aim was to estimate *μ*(*u*); the conditional expectation of the main effect of coherent motion as a function of position in the brain. We calculated *μ*(*u*) under the different priors above.

For demonstration purposes we selected a slice of the whole brain volume, which contained punctuate responses from bilateral posterior cingulate gyri (pCG). The conditional expectations under the Laplacian priors are shown in Fig. 5b for EGL and GGL. Although regions of high or low parameter estimates can be distinguished in the EGL analysis (left panel), the borders are difficult to make out. The posterior mean of GGL is different with well-delineated borders between regions of high and low coefficients, e.g., increased response in pCG. Activated regions are no longer ‘blobs’. This feature is easily seen in Fig. 5c with contour plot of a local kernel superimposed.

Posterior probability maps (Friston and Penny, 2003) of full slices for EGL and GGL are shown in Fig. 5d with thresholds *p*(*w* > 0.5) > 0.95 and close-ups of all three priors in Fig. 5e. The odd-one-out is the model with global shrinkage priors that had only one positive region within the whole slice. Surface plots are shown in Figs. 5f–h and graph embeddings in Figs. 5i and j. Note the vertical axes of surface plots showing largest magnitude for GGL and the degree of shrinkage with GSP.

The log-marginal likelihood for each model is shown in Table 2. The highest log-evidence was for GGL. The difference in log-evidence for the geodesic and Euclidean Laplacian priors was 260. This represents very strong evidence that the data were likely to be generated from a field with spatial covariance given by GGL compared to EGL. This sort of model comparison suggests that the data support the use of adaptive diffusion kernels when modeling spatial covariance of activation effects.

## Discussion

We have outlined a Bayesian scheme to estimate the optimal smoothing of conditional parameter estimates using a Gaussian process model. In this model, the prior covariance uses a diffusion kernel generated by a weighted graph Laplacian.

There are many issues to consider. We have only demonstrated the model using scalar-valued parameter images. A typical GLM of single subject data has a vector of parameters of size *P* × 1, at each voxel, with a covariance matrix, size *P* × *P*, where the design matrix has *P* columns. This means that there is a vector-field of regression coefficients over a brain volume. The weights of a GGL can be a function of scalars, vectors or matrices, which make it very flexible. For example, a GGL based on distance between parameter vectors at different voxels is easily implemented using the scheme presented here. More complex spaces, such as a field of symmetric positive definite (SPD) matrices, used to regularize Diffusion Tensor Images (DTI) (Chefd’hotel et al., 2002; Tschumperle and Deriche, 2003; Zhang and Hancock, 2006a), require methods from Lie group analysis, where a SPD matrix is represented as a sub-manifold of $R^{P^{2}}$. Matrices can be represented by vectors and probabilities over such a space can be represented by Gaussian densities (Begelfor and Werman, 2005), which suggests the possibility of using a Gaussian process prior over a spatial distribution of SPD matrices, or a Lie–Gaussian process prior. We also have considered the simplest noise model in this paper; however, noise models that vary over space, i.e., a heteroscedastic noise process, are also easily formulated using Gaussian process priors (see Chapter 5, Rasmussen and Williams, 2006) and Goldberg et al., 1998; Kersting et al., 2007). A possible use in fMRI is a GPP over autoregressive model coefficients in single subject data-sets following Penny et al. (2007).

A major computational issue is the time needed to compute the eigensystem of the Laplacian from which the matrix exponential, inverse, trace and determinant can be computed. The computational complexity scales with *N* ^3^, which is an issue for large data-sets. We have made no attempt to address this issue here, as our focus was on the combination of graph-theoretic approaches to image processing and spatial GPMs. The time taken to process the 3319 voxels in the random-effects analysis above was about 20 min on a standard personal computer. This has to be reduced, especially if a whole volume is to be processed. An advantage of a geometric formulation of the Laplacian is that 2D coordinates of the cortical surface can be used as the anatomical space and suggests using a cortical mesh, similar to that used in MEG source reconstruction. The cortical mesh is constructed from anatomical MRI and contains subject-specific anatomical information. A GPP based on such a diffusion kernel provides a way to formulate not only anatomically, but also functionally informed basis functions, thereby extending work by Kiebel et al. (2000).

There is a growing literature on sparse GPPs for regression (Lawrence, 2006; Quinonero-Candela and Rasmussen, 2005) used to formulate an approximate instead of a full GPP for use on large data-sets. Alternatively, multi-grid methods may be used (Larin, 2004) or we can utilize the graphical structure of the model and apply graph-theoretic methods to optimally partition a graph into sub-graphs or nested graphs (Tolliver et al., 2005). Recently, the computationally efficient properties of wavelets have been used to adaptively smooth fMRI parameter images (Flandin and Penny, 2007). Diffusion wavelets are an established method for fast implementation of general diffusive processes (Coifman and Maggioni, 2006; Maggioni and Mahadevan, 2006) and suggest an alternative implementation of the current method.

The issue of inverting large matrices is avoided by using the mean-field approximation of Penny et al. (2005). The spatial precision matrix they used is equivalent to the Euclidean graph Laplacian used here. This encodes local information, given by the neighborhood of a vertex, which they use in a variational Bayes scheme to estimate scaling parameters for each regressor, given data. The spatial covariance matrix can be calculated from the matrix exponential of this, which requires consideration when dealing with large data-sets as outlined above. What we get in return is a full spatial covariance that encodes global properties of the graph and the possibility of multivariate statistics over anatomical space.

As we focused on second level (between-subject) analyses the issue of temporal dynamics did not arise. However, for single-subject data this is not the case. A sensible approach would be to use a GLM to summarize temporal features in the signal and adaptively smooth over the vector-field of GLM regression coefficients, as mentioned above. Alternatively, a Kalman filter approach could be used; however, this may be more appropriate for EEG/MEG. The resulting algorithm would have a GPP for spatial signal with temporal covariance constrained by the Kalman filter.

The application of the current method to random-effects analysis was for demonstration purposes only. The method may also be useful in modeling functional and structural data from lesion studies, retinotopic mapping in fMRI, high-resolution fMRI and diffusion tensor imaging (Faugeras et al., 2004; Zhang and Hancock, 2006a).

## Figures and Tables

**Table 3:** Expressions used to compute log-marginal likelihood and its derivatives

A			
*Ã*_*k*_	*B˜*_*k*_		
∂*K*_1_/∂*υ*_1_	*S*_1_		
∂*K*_2_/∂*υ*_2_	*XS*_2_*X*^*T*^		
∂*K*_2_/∂*τ*	*XS*_2_*X*^*T*^		

B			

*A*_*k*_	*B*_*k*_	*C*_*k*_	*D*_*k*_

*K*_1_^− 1 ^*Ã*_*k*_	*S*_1_*^−1^B˜_k_*	*K*_1_^−1^*K¯K*_1_^− 1 ^*Ã*_*k*_	*S*_1_^− 1 ^*XS¯X*^*T*^*S*_1_^− ^1^^*B˜*_*k*_

**Table 1:** Model comparison for synthetic data shown in Fig. 3: fixed parameters and log evidence (natural logarithm) for EGL and GGL (difference shown in parentheses)

Covariance	Fixed parameters	Log evidence
EGL	*a*_1_ = *a*_2_ = 1	159.97
GGL	*a*_1_ = *a*_2_ = 1*a*_*μ*_ = 2	420.10 (260)

**Table 2:** Model comparison for synthetic (Fig. 4) and real data (Fig. 5)

Covariance	Fixed parameters	Synthetic data	Real data
GSP	*a*_1_ = *a*_2_ = 1	− 3.3325 × 10^3^	− 1.0371 × 10^5^
EGL	*a*_1_ = *a*_2_ = 1	− 3.0518 × 10^3^	− 1.0246 × 10^5^
GGL	*a*_1_ = *a*_2_ = 1*a*_*μ*_ = 2	− 2.9054 × 10^3^ (146)	− 1.0220 × 10^5^ (260)

Fixed parameters and log evidence for GSP, EGL and GGL (difference between GGL and EGL shown in parentheses).

## Appendix A Linear algebra for the **EM** scheme

This appendix provides notes on the linear algebra used to compute the gradients and curvatures necessary for the **EM** scheme in the main text. They are not necessary to understand the results presented above but help optimize implementation.

We require the bound on the log-marginal likelihood, ln *p*(*y*|*λ*)

(A.1) $$\begin{array}{ccc} F & = & -\frac{1}{2}(\ln|\Sigma |+y^{T}\Sigma^{-1}y+TN\ln2\pi )\\ \Sigma (\lambda ) & = & \Sigma_{1}+Z\Sigma_{2}Z^{T}\\ \Sigma_{i} & = & K_{i}⊗S_{i} \end{array}$$

and its derivatives. The first term of A.1 is

(A.2) $$\ln|\Sigma |=\ln|\Sigma_{1}|+\ln|\Sigma_{2}|+\ln|\Sigma_{2}^{-1}+Z^{T}\Sigma_{1}^{-1}Z|=\ln|\Sigma_{1}|+\ln|\Sigma_{2}|+\ln|\prod |$$

see Appendix of Rasmussen and Williams (2006). This can be reduced further using

(A.3) $$\ln|\Sigma_{i}|=\ln|K_{i}⊗S_{i}|=\mathrm{rank}(S_{i})\ln|K_{i}|+\mathrm{rank}(K_{i})\ln|S_{i}|$$

The second term of A.1 is

(A.4) $$\begin{array}{ccc} y^{T}\Sigma^{-1}y & = & tr(Y^{T}A)\\ A & = & S_{1}^{-1}{\hat{\varepsilon }}_{1}K_{1}^{-1} \end{array}$$

where we have used vec(*A*)^*T*^ vec(*B*) = *tr*(*A* ^*T*^ *B*) and *εˆ*_1_ = *Y* − *Xθˆ* is the matrix of prediction errors, where *μ* = vec(*θˆ*) (see Eq. (19)).

The conditional precision is

$$\prod =Z^{T}\Sigma_{1}^{-1}Z+\Sigma_{2}^{-1}=K_{1}^{-1}⊗X^{T}S_{1}^{-1}X+K_{2}^{-1}⊗S_{2}^{-1}$$

Given the number of columns, *P*, of the design matrix used in this paper is one (i.e., a scalar field of parameter estimates) and *K*_2_ includes a scale term, *υ*_2_, we can assume *X* ^*T*^ *S*_1_^− 1^ *X* = *S*_2_^− 1^ (note that this is not the case in general). The conditional precision then factorizes and we avoid computing the Kronecker tensor products implicit in the **EM** scheme. The precision then simplifies to

(A.5) $$\begin{array}{ccc} \prod & = & {\overset{―}{K}}^{-1}⊗{\overset{―}{S}}^{-1}\\ {\overset{―}{K}}^{-1} & = & K_{1}^{-1}+K_{2}^{-1}\\ {\overset{―}{S}}^{-1} & = & S_{2}^{-1} \end{array}$$

However, more generally, i.e., *P* > 1

(A.6) $$\begin{array}{ccc} \prod^{-1} & = & {\overset{―}{\Sigma }}_{1}-{\overset{―}{\Sigma }}_{1}V_{2}{\left(D_{2}+V_{2}^{T}{\overset{―}{\Sigma }}_{1}V_{2}\right)}^{-1}V_{2}^{T}{\overset{―}{\Sigma }}_{1}\\ {\overset{―}{\Sigma }}_{1} & = & K_{1}⊗{\left(X^{T}S_{1}^{-1}X\right)}^{-1}\\ V_{2} & = & V_{K_{2}}⊗V_{S_{2}}\\ D_{2} & = & D_{K_{2}}⊗D_{S_{2}} \end{array}$$

where we have used the matrix inversion lemma (see below), eigenvalue decomposition; *Σ*_2_ = *V*_2_ *D*_2_ *V*_2_^−^^1^ and

(A.7) $$C=A⊗B=(V_{A}⊗V_{B})(D_{A}⊗D_{B}){\left(V_{A}⊗V_{B}\right)}^{-1}$$

where *V* and *D* are eigenvectors and eigenvalues respectively of matrices A and B and in this case *V* ^−^^1^ = *V* ^*T*^. Using *S*_1_ = *I*_*T*_ and *S*_2_ = 1 the conditional mean decomposes into the OLS estimate and a shrinkage term due to the priors.

(A.8) $$\begin{array}{ccc} \mu & = & \mu_{\text{ols}}-P\mu_{\text{ols}}\\ P & = & {\overset{―}{\Sigma }}_{1}V_{2}{\left(D_{2}+V_{2}^{T}{\overset{―}{\Sigma }}_{1}V_{2}\right)}^{-1}V_{2}^{T}\\ \mu_{\text{ols}} & = & \text{vec}({\left(X^{T}X\right)}^{-1}X^{T}Y) \end{array}$$

To compute the derivatives required for the **M**-Step, we use the matrix inversion lemma (MIL)

(A.9) $$\begin{array}{ccc} {\left(\Sigma_{1}+Z\Sigma_{2}Z^{T}\right)}^{-1} & = & \Sigma_{1}^{-1}-\Sigma_{1}^{-1}Z{\left(\Sigma_{2}^{-1}+Z^{T}\Sigma_{1}^{-1}Z\right)}^{-1}Z^{T}\Sigma_{1}^{-1}\\ & = & \Sigma_{1}^{-1}-\Sigma_{1}^{-1}Z\prod^{-1}Z^{T}\Sigma_{1}^{-1} \end{array}$$

and standard results for Kronecker tensor products to show the score and expected information reduce to

(A.10) $$\begin{array}{ccc} \frac{\partial F}{\partial \lambda_{k}} & = & -\frac{1}{2}(tr(A_{k})tr(B_{k})-tr(C_{k})tr(D_{k})-tr({\tilde{A}}_{k}A^{T}{\tilde{B}}_{k}^{T}A))\\ I_{kl} & = & \frac{1}{2}tr((A_{k}⊗B_{k}-C_{k}⊗D_{k})(A_{l}⊗B_{l}-C_{l}⊗D_{l})) \end{array}$$

The second line can be simplified further using *tr*(*A⊗B*) = *tr*(*A*)*tr*(*B*). Expressions for *Ã*_*k*_, *B*˜_*k*_, *A*_*k*_, *B*_*k*_, *C*_*k*_, and *D*_*k*_ are given in Table 3
, where we have used *X* ^*T*^ *S*_1_^− 1^ *X* = *S*_2_^− 1^ (special case where *P* = 1).

## Appendix B Computing the model evidence; accuracy and complexity

Given A.2 and A.4 we can write the bound on the log-marginal likelihood as

(A.11) $$F=-\frac{1}{2}\left(\ln|\Sigma_{1}|+\ln|\Sigma_{2}|+\ln|\prod |+tr(Y^{T}A)+TN\ln2\pi \right)$$

After convergence of the **EM** scheme, this bound can be used as an approximation to the log-evidence, which can be expressed in terms of accuracy and complexity terms,

(A.12) $$F=-\frac{1}{2}\left(\ln|\Sigma_{1}|+{\hat{e}}_{1}^{T}\Sigma_{1}^{-1}{\hat{e}}_{1}+TN\ln2\pi +\ln|\Sigma_{2}|+\ln|\prod |+\mu^{T}\Sigma_{2}^{-1}\mu \right)$$

where *ê*_1_ = vec(*εˆ*_1_). The first three terms of A.12 represent the accuracy and remaining terms complexity. To see the equivalence of A.11 and A.12 more clearly,

$$\begin{array}{ccc} y^{T}\Sigma^{-1}y & = & y^{T}\Sigma_{1}^{-1}y-\mu^{T}\prod \mu \\ & = & y^{T}\Sigma_{1}^{-1}y-2\mu^{T}\prod \mu +\mu^{T}\prod \mu \\ & = & y^{T}\Sigma_{1}^{-1}y-2\mu^{T}Z^{T}\Sigma_{1}^{-1}y+\mu^{T}Z^{T}\Sigma_{1}^{-1}Z\mu +\mu^{T}\Sigma_{2}^{-1}\mu \\ & = & {\left(y-Z\mu \right)}^{T}\Sigma_{1}^{-1}(y-Z\mu )+\mu^{T}\Sigma_{2}^{-1}\mu \end{array}$$

where the MIL and Eq. (19) are used in the first line and Eq. (19) again in the second. To see the decomposition into accuracy and complexity terms consider the likelihood, prior and posterior of the parameters (*p*(*y*|*w*,*λ*), *p*(*w*) and *q*(*w*) respectively), note that

(A.13) $$\begin{array}{ccc} F(q,\lambda ) & = & \int dwq(w)\ln p(y|w\text{,}\lambda )-\int dwq(w)\ln\frac{q(w)}{p(w)}\\ & = & {\langle L(w)\rangle }_{q(w)}-\mathrm{KL}(q(w)||p(w))\\ {\langle L(w)\rangle }_{q(w)} & = & -\frac{1}{2}(\ln|\Sigma_{1}|+{\hat{e}}_{1}^{T}\Sigma_{1}^{-1}{\hat{e}}_{1}+tr(Z^{T}\Sigma_{1}^{-1}Z\prod^{-1}))\\ \mathrm{KL}(q||p) & = & \frac{1}{2}(\ln|\Sigma_{2}\prod |+\mu^{T}\Sigma_{2}^{-1}\mu +tr(\Sigma_{2}^{-1}\prod^{-1})-tr(I_{PN})) \end{array}$$

where 〈*L*(*w*)〉_*q*(*w*)_ is the average log-likelihood under *q*(*w*) and the Kullback–Leibler (KL) divergence is a penalty term on the parameters. We have used standard results for Gaussian densities *N*(*w*; *m*, *S*)

(A.14) $$\begin{array}{c} \int dx{\left(\mu -Wx\right)}^{T}\Sigma^{-1}(\mu -Wx)N(m\text{,}S)={\left(\mu -Wm\right)}^{T}\Sigma^{-1}(\mu -Wm)+tr(W^{T}\Sigma^{-1}WS)\\ \mathrm{KL}(q||p)=\frac{1}{2}\ln|\Sigma_{1}\Sigma_{0}^{-1}|+\frac{1}{2}tr(\Sigma_{1}^{-1}((\mu_{0}-\mu_{1}){\left(\mu_{0}-\mu_{1}\right)}^{T}+\Sigma_{0}-\Sigma_{1}) \end{array}$$

where the KL divergence is over two Gaussian densities *q*(*w*) ∼ *N*(*μ*_0_, *Σ*_0_) and *p*(*w*) ∼ *N*(*μ*_1_, *Σ*_1_), i.e., are the posterior and prior densities over the parameters.

In practice, optimization of non-negative scale parameters in the **M**-Step uses the transform; *γ*_*i*_ = ln*λ*_*i*_. The derivatives in Table 3 are then ∂*K*/∂*γ*_*i*_ = *λ*_*i*_∂*K*/∂*λ*_*i*_. Under this change of variables, the hyperparameters have non-informative log-normal hyper-priors. Uncertainty about the hyperparameters can be included in the log-evidence for any model *m*. For example, the approximate log-evidence including uncertainty of one hyperparameter is

(A.15) $$\ln p(y|m)\approx \ln p(y|\gamma \text{,}m)-\frac{1}{2}\ln\frac{\partial^{2}F}{\partial \gamma^{2}}=\ln p(y|\gamma \text{,}m)+\frac{1}{2}\ln I$$

Where the second-order derivative is the expected information used in the **M**-Step. See Friston et al. (2007) for details.

## Appendix C Metrics on manifolds

The intuition behind the induced metric comes from considering Pythagoras' theorem. See Fig. 1c (inset).

(A.16) $$ds^{2}=du^{2}+ad\mu^{2}=\left(1+a{\left(\frac{d\mu }{du}\right)}^{2}\right)du^{2}=Gdu^{2}$$

More formally, consider a one-dimensional curve embedded in two-dimensional Euclidean space. A map from one manifold, (*M*, *g*), to another, (*N*, *h*), where *G* and *H* are metrics associate with each respectively, is

(A.17) $$\begin{array}{c} \chi :M\to N\\ \chi :u\to (\chi^{1}(u)\text{,}\chi^{2}(u))=(u\text{,}\mu (u)) \end{array}$$

Where *u* is a local coordinate on the curve and *χ* ^1^ and *χ* ^2^ are coordinates in the embedding space. A distance *d*_*s*_ on the curve in terms of d*u* is given by

(A.18) $$\begin{array}{ccc} H & = & \left(\begin{array}{cc} 1 & 0\\ 0 & a \end{array}\right)\\ J & = & \frac{\partial \chi }{\partial u}=\left(\begin{array}{c} 1\\ \mu_{u} \end{array}\right)\\ G & = & J^{T}HJ=(1\;\mu_{u})\left(\begin{array}{cc} 1 & 0\\ 0 & a \end{array}\right)\left(\begin{array}{c} 1\\ \mu_{u} \end{array}\right)\\ & = & 1+a\mu_{u}^{2}\\ d_{s}^{2} & = & Gdu^{2} \end{array}$$

Where the relative scale between the domain and feature coordinates is *a* and *G* is the induced metric, i.e., metric on the curve.

## Appendix D Computing the graph Laplacian

We assemble the graph Laplacian using a 3 × 3 stencil with 8 nearest neighbors. See Fig. 1 showing how the distance between function values (parameter image) at different points in the embedded sub-manifold are represented by a graph with edge weights, *w*_*kn*_. Computing the eigensystem of the graph Laplacian simplifies many computations; for example

(A.19) $$L=V\Lambda V^{T}\text{diag}(\Lambda )=(\lambda_{0}\text{,}\lambda_{1}\text{,}\ldots \text{,}\lambda_{N-1})\mathrm{, where}\;\lambda_{i}\geq 0K=V\exp(-\Lambda )V^{T}K^{-1}=V\exp(\Lambda )V^{T}\left|K|=\prod_{i=0}^{N-1}\exp(-\lambda_{i}\right)tr(K)=\Sigma_{i=0}^{N-1}\exp(-\lambda_{i})$$

The third line affords a way to compute the matrix exponential (Moler and Van Loan, 2003). The issue with updating the graph Laplacian with each iteration based on the posterior mean of the parameters is that it entails a composition of non-commuting matrices. An approximation is possible using a truncation of the Baker–Campbell–Hausdoff (BCH) formula (Rossmann, 2002).

(A.20) $$\begin{array}{ccc} K^{\left(m\right)} & = & \exp(Q^{\left(m\right)})\\ K^{\left(m+1\right)} & = & \exp(-L^{\left(m\right)}\tau^{\left(m+1\right)})K^{\left(m\right)}\\ & = & \exp(Q^{\left(m+1\right)})\\ Q^{\left(m+1\right)} & = & Q^{\left(m\right)}+dQ^{\left(m+1\right)}\\ dQ^{\left(m+1\right)} & \approx & -(L^{\left(m\right)}\tau^{\left(m+1\right)}+\frac{1}{2}[L^{\left(m\right)}\tau^{\left(m+1\right)}\text{,}Q^{\left(m\right)}]) \end{array}$$

where we have used the first three terms of the BCH formula;

(A.21) $$\begin{array}{ccc} C & = & \log(\exp(A)\exp(B))\\ C & \approx & A+B+\frac{1}{2}[A\text{,}B] \end{array}$$

[*A*, *B*] is the commutator of operators, *A* and *B* given by [*A*, *B*] = *AB* − *BA*.

## Footnotes

1 Element-wise exponential as opposed to matrix exponential used in Eq. (6).

2 This means that the vectorized random matrix has a multivariate normal density, given by vec(*A^T^*) ∼ *N*(vec(*M^T^*),*S*⊗*K*) and vec(*A*) ∼ *N*(vec(*M*),*K*⊗*S*).

3 Minus sign used by convention.

4 This is equivalent to a Newton step, but using the expected curvature as opposed to the local curvature of the objective function.
